# The use of routine data from primary care practices in Germany to analyze the impact of the outbreak of SARS-CoV-2 on the utilization of primary care services for patients with type 2 diabetes mellitus

**DOI:** 10.1186/s12875-022-01945-y

**Published:** 2022-12-16

**Authors:** Christoph Strumann, Paul-Georg Blickle, Wolfgang C. G. von Meißner, Jost Steinhäuser

**Affiliations:** 1 Institute of Family Medicine, University Hospital Schleswig-Holstein, Campus Luebeck, Ratzeburger Allee 160, 23538 Luebeck, Germany; 2 Hausärzte am Spritzenhaus, Family Practice, Baiersbronn, Germany

**Keywords:** Diabetes mellitus, Routine data, SARS-CoV-2, Covid-19, Primary care, Germany

## Abstract

**Background:**

Routinely collected health data from ambulatory care providers offer a wide range of research opportunities. However, the access is often (e.g., technically) hindered, particularly in Germany. In the following, we describe the development of an infrastructure for the analysis of pseudonymized routine data extracted from primary care practices in Germany. Further, we analyze the impact of the outbreak of SARS-CoV-2 on the utilization of primary care services for patients with type 2 diabetes mellitus (DM type 2).

**Methods:**

In this retrospective cohort study, routine data were extracted from nine private primary care practices before and since the outbreak of SARS-CoV-2 in Germany. The sample consisted of patients who were treated between 2016 and 2022 in one of the participating practices. The effects of the outbreak on the frequency of practice visits and the disease course of DM type 2 patients were analyzed by means of bivariate and multivariate analyses.

**Results:**

The developed infrastructure offers an analysis of routine data from outpatient care within 24 h. In total, routine data of 30,734 patients could be processed for the analyses with 4182 (13.6%) patients having a diagnosed DM type 2 and 59.0% of these patients were enrolled in a disease management program (DMP). In the multivariate analysis, there was a significant negative effect of the SARS-CoV-2 outbreak on utilization of outpatient services of patients with DM type 2 disease. This decrease was less pronounced among DMP patients. The glycated haemoglobin level (HbA1c) has not changed significantly.

**Conclusions:**

The study showed that the analysis of routine data from outpatient care in Germany is possible in a timely manner using a special developed electronic health record system and corresponding software. The significantly negative effect of the SARS-CoV-2 outbreak on utilization of outpatient services of patients with DM type 2 disease was less pronounced among DMP patients. Two years after the start of the Covid pandemic a significantly worsened course of illness cannot be observed. However, it must be taken into account that the observation period for clinically relevant outcomes is still relatively short.

**Supplementary Information:**

The online version contains supplementary material available at 10.1186/s12875-022-01945-y.

## Background

Routinely collected health data offer a wide range of research opportunities including epidemiological studies [1], technology-assessment [2], and the evaluation and improvement of care [3]. Since today most health care records are stored electronically, the access to routine data in research has been massively simplified in most countries [4]. The collection is considered as less cost intensive while covering a large size of the population and, thus, comprise a higher representativeness [5].

Increased access to routine data for research is often hindered by privacy and ethical concerns about the secondary use of these data [3], particularly in Germany [6]. Despite these concerns, Germany has well-defined infrastructures for accessing routine data from hospitals. In 2011, for instance, the Healthcare Structure Act (“Versorgungsstrukturgesetz”) facilitated access to the performance data of the health insurance funds. The German Institute for Medical Documentation and Information (DIMDI) has built a database of pseudonymized claims data, which has been assessable for selected stakeholders for research purposes since February 2014. This has led to routine data from hospitals being used extensively in research [7].

In addition to these developments, the Federal Joint Committee (G-BA, mandated to make legally binding decisions on the entitlement to benefits of people with statutory health insurance in Germany) has encouraged the evaluation of health services to be based on routine data analysis, e.g. to form the basis for estimating the need for inpatient treatment or morbidity [8]. Regarding routine data from ambulatory care providers, however, there is no such an infrastructure. Since the German ambulatory care sector is highly separated from inpatient care and is organized in mostly privately run practices under free provider choice [9], there is a wide range of applied electronic health record systems (EHRs) that are largely inadequate to extract data [10, 11]. Several feasibility studies have shown how the use of routine data for ambulatory care would be technically possible [12, 13], but are hindered by outdated software interfaces, insufficient software maintenance, organizational and financial burdens imposed by software vendors, and deficient IT standards [6].

The routine data available for research in Germany are often only available with a long delay. This circumstance also revealed the problem in the Corona pandemic that no current care data have been available in Germany. In contrast, e.g., to the registry on intensive care bed occupancy of the German Interdisciplinary Association for Intensive Care and Emergency Medicine (DIVI) [14], similar monitoring for the outpatient setting was lacking, although about 85% of the patients infected with SARS-CoV-2 were predominantly cared for by family physicians [15]. Access to current data from primary care physicians would therefore be helpful in many respects for the assessment of measures, e.g., during the course of a pandemic.

In this analysis, we describe the development of an infrastructure for the analysis of pseudonymized routine data extracted from the EHRs of nine private primary care practices (eight operating facilities) in the federal state of Baden-Württemberg in the south-west of Germany. Based on these data we analyze the impact of the outbreak of SARS-CoV-2 on the utilization of primary care services for patients with type 2 diabetes mellitus (DM type 2). Different analyses have shown that there is a reduced utilization behavior due to the Covid pandemic [16, 17]. As a consequence, patients seeking medical help, e.g. in emergencies, are characterized by higher severity [18]. The decline in physician contacts is particularly pronounced among potential risk groups [19], for those a continuity of care is of particular importance to quality of care and quality of life [20]. With an increasing prevalence of 8.5% in 2009 to 9.5% in 2015, diabetes mellitus type 2 is one of the most common chronic diseases in Germany [21]. Patients require continuous primary care to monitor blood glucose metabolism and minimize diabetic sequelae. Specific disease management programs (DMPs) consisting of a system of coordinated health care interventions and information for chronically ill patients are designed to foster active patient participation in their treatment. Introduced in 2002, the DMP on “diabetes mellitus type 2” aims to treat patients with diabetes according to evidence-based guideline standards under shared decision-making conditions. In pro-active doctor-patient appointments every three month in the primary care practice, the health goals are defined, and its attainment are discussed. Patients need to consent for the enrolment and recall list. Clinically, diabetic related complications are screened within these appointments (e.g., diabetic foot syndrome, diabetic nephropathy) [22]. Whereas screening regarding diabetic retinopathy is performed once a year in an ophthalmological practice. This regular exchange is part of the continuous medical support over the different phases of life and the management of the chronic disease. The structured treatment is intended to improve the quality of care [22, 23]. For the enrolment of (statutorily insured) patients, physicians are rewarded by additional payments. Financial incentives are also offered by German sickness funds to patients participating in DMPs [24].

Against this background, we analyze potential pandemic-related changes in outpatient care for patients with type 2 diabetes mellitus in Germany based on a newly developed unique database consisting of pseudonymized routine primary care data. Further, we analyze the role of the DMP for the care of patients with a DM type 2 during the Covid-19 pandemic.

## Methods

### Data source

The study is a retrospective cohort study of routine data extracted from nine private practices in ambulatory care before and since the outbreak of SARS-CoV-2 in Germany. The participating practices were recruited from the MEDI-Verbund Baden-Württemberg e.V. (Health Services Research Network, HSRN). In these practices, 38 general practitioners are treating annually more than 100,000 cases. In addition to sociodemographic information, data about the practice visits (diagnoses and prescriptions), laboratory test results and data about the examinations of the DMPs were stored in the EHRs of the practices. The sample consisted of patients who were treated during the observed period (2016q1-2022q1) in one of the participating practices.

### Evaluation content and study variables

This analysis examined two different aspects of the consequences of a potential change in outpatient utilization patterns due to the SARS-CoV-2 outbreak. First, the effects of the outbreak on the utilization of outpatient services for DM type 2 patients were considered. Second, data providing information on the disease course of patients with a chronic disease were analyzed. The observation unit was one patient with DM type 2 from one of the participating practices of the MEDI HSRN per quarter (three-month period) of a year (first quarter of 2016 to first quarter of 2022).

The primary study objective was to quantify the effect of the SARS-CoV-2 outbreak on outpatient service utilization. For this purpose, the number of practice visits, number of referrals and inpatient admissions of a patient in a quarter were considered as outcome variables. A practice visit was defined if there was a documentation from the treating physician stored in the EHR or a corresponding claim was billed. Referrals and admissions were documented accordingly in the EHR.

The health status was considered as a secondary outcome. For this purpose, blood pressure (RR systolic and RR diastolic) and the number of diseases were used. The latter was operationalized by the number of ICD diagnoses from different chapters. Further, the body mass index (BMI) and diabetes specific outcomes as the HbA1c-value and the metabolic derailment (based on respective ICD diagnoses) were considered as secondary outcomes.

### Data processing

**Fig. 1:**
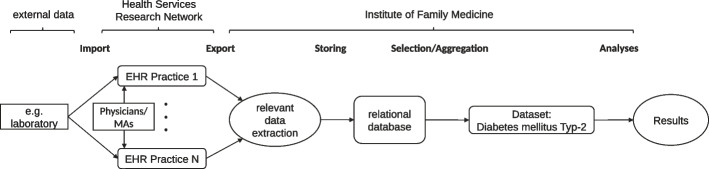
Data Flow Chart

Figure 1 shows the flow of the data processing from the import to the HER to the cleaned dataset used for the statistical analysis. In each of the participating MEDI practices, external data (e.g., laboratory findings as HbA1c) are imported routinely into the EHRs. Master and case data are entered into the respective EHR by the physicians or medical assistants (MAs). These routine data can be sent pseudonymized as an export from each practice to the Institute of Family Medicine in Lübeck. Subsequently, the characteristics relevant for further analyses will be automatically extracted from the exported data by means of a self-developed software tool. The extracted data were stored in a relational database in a structured manner. From this database, a specific data set was prepared according to the requirements for the current analysis.

### Technical implementation of importing and exporting EHR data

The practice management system (abasoft EVA (Electronically Managed Medical Practice) version 239) of the participating practices in the MEDI HSRN has suitable interfaces that enable automated import of external data and same-day export of routine data. External data can be imported as a CSV (comma-separated values) import file into the EHR of each practice via an interface. This enables a transfer of externally collected values to the patient record. In order to assign the external data to the correct patient in the EHR database, the EHR compares the last name, first name and date of birth from the CSV import file with the existing patient master data in the EHR of the practice. If these three parameters are identified in the EHR database, the data from the CSV import file is transferred to the file of the respective patient. The patient's first and last names must be written exactly with the correct capital and lower-case letters as well as special characters in order to obtain a match in the EHR database. If these three parameters are not identified, a new patient is created in the EHR database with the data stored in the CSV import file and a new consecutive and unique patient identification number.

The CSV import file is coded according to ISO 8859–1 and defined as follows:

- The data to be imported are divided into master data and case record data.
- **Master data:** data that remain unchanged over a longer period of time or permanently and are stored in the EHR in the master data mask. This includes, e.g., personal data such as last name, first name, gender, street with house number, postal code, city, insurance number (institute code of the health insurance company), insured person number (membership number), membership status, telephone number, mobile phone number, 2nd telephone number, e-mail address and identification of the referring physician or general practitioner (site number (BSNR) and lifetime physician number (LANR)).
- **Case record data**: data on the course of treatment. These data are stored in different text groups, some of which can be individually defined in the EHR. Uniformly defined text groups exist, e.g., for acute diagnoses, permanent diagnoses and text groups for billing codes. Individual text groups exist, e.g., for anamnesis, medical findings, social anamnesis, family anamnesis, risk factors, procedures, therapy, caution, allergies, sonography findings, archive entries, etc. Case record data can be written to any defined text group via the CSV import file. The text group entry is limited to 64 characters (screen width in the EHR case record view). To write multi-line entries to the case record, multi-line CSV import files must be created.

Established standard interfaces are used to import external data into the EHR. These interfaces exist in Germany since the 1980s and became mandatory in the 1990s [25]. Laboratory values are imported via these interfaces [Labor-Datenfernübertragung-Interface] (LDT). Medical devices such as ECG and ultrasound systems communicate via Gerätedatentransfer (GDT) interface [26]. These interfaces are very limited and restricted to their specifications [11].

### Export of routine data from the EHR

The master and case record data stored in the EHR can be identified and exported using a patient list search. In the administration of the patient list search in the EHR, logic definitions can be created for search runs and thus search queries can be defined. Up to 16 conditions can be defined and linked with each other. Search queries are possible in any text groups and text group combinations as well as in the master data, e.g., health insurance company and place of residence. These search queries can be limited quantitatively as well as temporally and combined with the logic parameters "and" and "and/or" as well as their negation with any bracket rules.

For the evaluation of data, these patient lists can be marked for export in CSV format at a fixed time. This setting can also be specified for certain days of the week and times enabling a regular automated data export.

The export was performed pseudonymously by exporting the practice-internal patient identification number and replacing the personal data (last name, first name, street address, date of birth, etc.) from the master data with a *. Previously to the pseudonomization, the month and the year of the date of birth were extracted and saved in the export file. The exported routine data were then made available (retroactively from 2016) to the Institute of Family Medicine in Lübeck for further data processing.

### Software tool to extract the routine data and generate the data set for the analyses

A software program was developed at the Institute of Family Medicine to extract the relevant data and to store the data anonymously in a relational database in a structured manner. In a first step, the data were extracted patient by patient for each year. For each patient in a specific year, all entries were searched for relevant content in the defined and individual text groups. In a second step, the entries were structured and stored in separate tables, which can be assigned to the following subjects:

- master data (e.g., gender, insurance status, etc.)
- medical history and findings data collected during a consultation (e.g., examinations, blood pressure measurements, etc.)
- prescription data
- billing codes
- diagnoses
- laboratory results

In a third step, all extracted entries were checked for duplicates. Duplicates are entries identified as identical for the same patient at the same time. Duplicates were deleted. Finally, the tables were stored in a relational database, which contains all considered years of all practices.

### Statistical analysis

The statistical analyses were performed at the quarterly level and for patients with type 2 diabetes mellitus. For this purpose, a suitable data set was created based on the tables stored in the relational database. The considered patients were selected with regard to information from the diagnosis data and the billing figures used for DMP documentation [27]. Subsequently, the relevant information for each patient was cleaned and aggregated in a suitable manner at the quarterly level (e.g., number of physician visits per quarter).

Since there was no control group, but all patients were simultaneously affected by the general contact restrictions due to the outbreak of SARS-CoV-2, the effects of the outbreak on the various outcome variables were determined using a before-after comparison in a bivariate and a multivariate analysis. In the bivariate analysis, the primary outcomes measuring the service utilization and the state of health (secondary outcomes) were compared before and after the pandemic. Differences of the considered variables were tested by means of t-test or χ^2^-test (if the respective variable is nominally scaled).

The multivariate regression analysis concentrates on the number of practice visits and the glycated hemoglobin level (HbA1c value (%)). For both considered outcome variables, the following generalized linear model was applied

$$E\left[ {y}_{ijt} | {{\varvec{x}}}_{it-1}^{^{\prime}}\right] = {g}^{-1}(\delta I\left(t>2020q1\right)+{\sum }_{p=1}^{P}{\alpha }_{p}{t}^{p}+{\sum }_{k=1}^{K}{\lambda }_{k}{q}_{k}+ {{\varvec{x}}}_{it-1}^{^{\prime}}\beta +{\mu }_{i}+{\nu }_{j}),$$

where ${y}_{ijt}$ is the corresponding outcome variable of patient $i$ in practice $j$ at time $t\in \{2016{q}_{1},\dots ,2022{q}_{1}\}$, $g$ is a link function, the indicator function $I\left(t>2020q1\right)=1$, if $t>2020q1$ and $\delta$ measures the effect of the SARS-CoV-2 outbreak on ${y}_{ijt}$. To minimize the risk of a biased estimate for $\delta$ due to potential confounding variables, we specified a general time trend (${\sum }_{p=1}^{P}{\alpha }_{p}{t}^{p}$) as well as seasonal effects (${\sum }_{k=1}^{K}{\lambda }_{k}{q}_{k}$). Moreover, we controlled for time-invariant heterogeneity among the patients (e.g., gender, individual susceptibility to disease/family history) and the practices (e.g., location, equipment) by specifying corresponding individual effects (${\mu }_{i}$ and ${\nu }_{j}$). In addition to gender, time-varying patient-level control variables from the previous quarter (${{\varvec{x}}}_{it-1}^{^{\prime}}$), such as the number of prescriptions and diagnosis groups (ICD-chapters) from practice visits, were also included. The polynomial of the trend specification $P$ was selected by the Bayesian-Information-Criterion (BIC). To counteract possible dependencies of the effects by the trend specification, the robustness of the results was examined in sensitivity analyses with various values of $P$.

Data analysis was performed using MATLAB R2021a and STATA 15.

### Identification of patients

In some practices, corona tests were also performed for patients who were not originally cared for by the general practitioner of the practice. However, the data of these patients were integrated into the respective EHR. Further, due to the trans-regional offer for corona vaccination of some practices, data of vaccinated patients were also deposited in the EHR, even if they were cared for by another practice. The data of these patients were excluded from the analysis. More precisely, all patients for whom no practice visit was deposited in the HER prior to the SARS-CoV-2 outbreak and who visited the practice after the outbreak for corona vaccination or because of a corona test were not considered for this analysis. To examine the impact of the SARS-CoV-2 outbreak on patient utilization and course of disease, patients who did not have data for the respective outcome in at least one quarter before and after the outbreak were excluded.

## Results

### Sample characteristics

Table 1 shows the total number of patients in the individual practices and the number of patients for whom a DM type 2 was documented during the sample period. In addition, the number of those who were enrolled in a corresponding DMP is tabulated for the individual practices.

**Table 1:** Number of patients, n (%)

Practice-ID	total	DM type 2		
		total	DMP*	Mulitmorbid*^a^
1	7864	938 (11.9)	520 (55.4)	402 (42.9)
2	7336	913 (12.4)	515 (56.4)	414 (45.3)
3	3744	597 (15.9)	380 (63.7)	336 (56.3)
4	3483	518 (14.9)	320 (61.8)	258 (49.8)
5	2787	373 (13.4)	229 (61.4)	220 (59.0)
6	2008	321 (16.0)	206 (64.2)	154 (48.0)
7	1259	192 (15.3)	97 (50.5)	93 (48.4)
8	2253	330 (14.6)	200 (60.6)	184 (55.8)
total	30,734	4182 (13.6)	2467 (59.0)	2061 (49.3)

^*^The proportions (%) refer to the number of patients with a diagnosed DM type 2 disease

^a^Multimorbid patients were identified, if more than two chronical diagnoses [28] were coded in at least 3 quarters within the sample period [29]

In total, routine data of 30,734 patients could be processed for the analyses. Of these, 4182 (13.6%) had DM type 2 with a proportion of DMP patients of 59.0% and multimorbid patients of 49.3%.

The average age of the total patients in the first quarter of 2020 was 51.6 years, while patients with a DM type 2 were approximately 18 years older (Table 2). While the majority of the total patients were female, the gender distribution among the patients with a DM type 2 were rather equal. Patients with a DM type 2 and enrolled in a DMP were on average 70 years old and in 50.5% female. The patients suffering from DM type 2 had on average 1.37 to 1.71 (DMP group) practice visits per quarter during the entire observation period and were referred to another practice 0.86 to 0.78 (DMP group) times. The number of hospitalizations per quarter was 0.01 to 0.02 (DMP group).

**Table 2:** Characteristics of Patients with a DM type 2 per quarter, mean/N

	total	DM type 2	
		all	DMP
2016q1-2022q1	N = 755,776 (30,734 patients)	N = 100,518 (4182 patients)	N = 59,309 (2467 patients)
Age (2020q1)	51.6	69.3	70.0
Female, n (%)	417,424 (55.2)	51,011 (50.7)	29,952 (50.5)
Male, n (%)	338,089 (44.7)	49,494 (49.2)	29,357 (49.5)
Transgender/diverse, n (%)	38 (0.0)	13 (0.0)	0 (0.0)
Number of prescribed medications	1.6	5.0	6.5
BMI (kg/m^2^)	28.4/57100*	30.26/25361*	30.36/21506*
*Outcomes*			
Number of practice visits	0.68	1.37	1.71
Number of referrals	0.25	0.58	0.78
Number of hospital admissions/referrals	0.01	0.01	0.02
RR-Systolic (mmHg)	135.1/41024*	137.8/16486*	137.9/13268*
RR-Diastolic (mmHg)	79.6/40911*	79.0/16428*	78.8/13219*
Number of diagnoses at practice visit (ICD-Chapter)	1.95	2.15	2.15

First Quarter: q1; N: number of observations; *Missing values due to non-performed examination or no practice contact

^a^Multimorbid patients were identified, if more than two chronical diagnoses [28] were coded in at least 3 quarters within the sample period [29]

### Development of utilization and health status

**Fig. 2:**
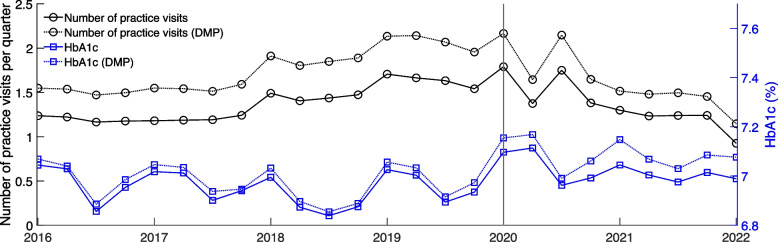
Number of practice visits and HbA1c (%) per quarter

Figure 2 shows the development of the number of practice visits of all patients with a DM type 2 disease per quarter and the HbA1c of these patients over time before and after the outbreak of SARS-CoV-2. While before 2018 the average number of practice visits for DM type 2 patients was just over 1.2 per quarter, it increased slightly to 1.7 visits per quarter by the beginning of 2019 (see Table S1 in the Additional file 1). In the first quarter of 2020, there was an increase to 1.79 visits. In contrast, after the pandemic outbreak, the average number of contacts dropped to 1.4, followed by a somewhat weaker rebound. Beginning in the third quarter of 2020, practice visits steadily declined, reaching a level of 0.9 in the first quarter of 2022. For patients who were enrolled in the DMP, the observed trends are very similar, with higher levels and a stronger decline due to the outbreak of SARS-CoV-2.

The HbA1c value shows a strong seasonality before the outbreak of SARS-CoV-2. While the value reaches a minimum in the third quarter, it is highest in the first quarter. After the pandemic outbreak, the seasonal pattern shifts by a generally slightly higher level. In the second quarter of 2020, the HbA1c value reaches a maximum, followed by a minimum in the third quarter of 2020. In the first quarter of 2022, contrary to the pre-pandemic pattern, there was a slight decrease from the previous quarter. DM type 2 patients enrolled in the DMP generally have higher HbA1c values. This difference became slightly larger after the SARS-CoV-2 outbreak.

#### Bivariate analysis

Before the pandemic, all DM type 2 patients had an average of 1.33 office contacts per quarter (Table 3) that decreased by 1.2% to 1.31 since the SARS-CoV-2 outbreak. However, this change is similar to the changes of the number of referrals as well as hospital admissions not significant. Similarly, changes in the BMI cannot be observed. The measures of the blood pressure both increased by 2.8% (systolic) and 1.7% (diastolic) as well as the HbA1c value increased from 6.96 to 7.04 (1.2%). The occurrence of metabolic derailments remained unchanged.

**Table 3:** Comparison of pre- and post-outbreak measures for patients with DM type 2

	Number of patients	Pre-outbreak 2016q1-2020q1		Post-outbreak 2020q2-2022q1		Difference	
		N	μ	N	μ	Δ (%)	*p*-value
Number of practice visits	3957	67,269	1.33	30,880	1.31	-1.2%	0.2711
Number of referrals	3957	67,269	0.57	30,880	0.59	2.6%	0.0607
Number of hospital admissions/referrals	3957	67,269	0.01	30,880	0.01	10.3%	0.2116
BMI (kg/m^2^)	1990	11,935	30.3	9511	30.3	-0.1%	0.8188
RR-Systolic (mmHg)	1314	4399	135.6	6579	139.4	2.8%	< 0.001
RR-Diastolic (mmHg)	1311	4348	78.4	6568	79.7	1.7%	< 0.001
Number of diagnoses at practice visit (ICD-Chapter)	3266	33,369	2.10	21,387	2.07	-1.5%	0.047
*Diabetic-specific outcome*							
Metabolic derailment (yes, %)	3957	67,269	0.97	30,880	1.04	7.9%	0.2612^b^
HbA1c (%)	2040	14,518	6.96	9280	7.04	1.2%	< 0.001

First Quarter: q1

^a^Number of patients with non-missing data for the variable under consideration; μ: mean; N: Number of observations; Δ (%): relative difference between before and after the pandemic

^b^χ^2^ Test

Table 4 shows the same analysis for patients with DM type 2 that were enrolled in the DMP. Practice visits and referrals decreased more strongly as the total group of DM type 2 patients by 7.3% and 3.8%, respectively. Hospital admissions did not change. The variables measuring the health status/development (blood pressure, HbA1c value, etc.) show not a different development as before.

**Table 4:** Comparison of pre- and post-outbreak measures for patients with DM type 2 enrolled in the DMP

	Number of patients	Pre-outbreak 2016q1-2020q1		Post-outbreak 2020q2-2022q1		Difference	
		N	μ	N	μ	Δ (%)	*p*-value
Number of practice visits	2333	39,661	1.69	18,218	1.57	-7.3%	< 0.001
Number of referrals	2333	39,661	0.78	18,218	0.75	-3.8%	0.0121
Number of hospital admissions/referrals	2333	39,661	0.02	18,218	0.01	-13.3%	0.1498
BMI (kg/m^2^)	1612	10,540	30.4	8390	30.4	0.0%	0.9706
RR-Systolic (mmHg)	1024	3686	135.8	5682	139.3	2.6%	< 0.001
RR-Diastolic (mmHg)	1022	3640	78.2	5672	79.7	1.9%	< 0.001
Number of diagnoses at practice visit (ICD-Chapter)	2095	25,195	2.10	15,041	2.11	0.3%	0.703
*Diabetic-specific outcome*							
Metabolic derailment (yes, %)	2333	39,661	1.30	18,218	1.43	9.9%	0.2111
HbA1c (%)	1603	12,953	6.98	8012	7.08	1.5%	< 0.001

First Quarter: q1

^a^Number of patients with non-missing data for the variable under consideration; μ: mean; N: Number of observations; Δ (%): relative difference between before and after the pandemic

^b^χ^2^ Test

#### Multivariate analysis

Table 5 shows the estimated effects of the outbreak of SARS-CoV-2 on the number of practice visits and on the HbA1c-value taken into account for control variables as well as individual effects at the patient and practice level. Based on the model selection criteria (BIC), the polynomial of the trend specification was selected for the number of practice visits as $P=8$ and for the HbA1c-value as $P=3$. The regression results show a decrease in practice contacts by 12.2% ($-0.122$, $p<0.01$). The positive interaction effect between the pandemic outbreak and DMP ($0.092$, $p<0.01$) suggests that, in contrast to the results of the bivariate analyses, the decrease in practice contacts is significantly lower for DMP patients. Further, no significant effect of the pandemic on the HbA1c-value can be found in the multivariate analysis.

**Table 5:** Multivariate analysis of the Covid-Outbreak

Variable	Utilization: Number of practice visits		Course of the disease: HbA1c	
Trend specification:$P$	8	8	3	3
Quarter (Reference: Quarter 1)				
Quarter 2	-0.023*	-0.023*	-0.060***	-0.060***
Quarter 3	0.029***	0.030***	-0.134***	-0.135***
Quarter 4	0.003	0.003	-0.097***	-0.097***
Covid-Outbreak ($\delta$)	-0.122***	-0.189***	-0.028	-0.001
DMP	0.450***	0.407***	0.303***	0.318***
Covid- Outbreak ($\delta$) X DMP		0.092***		-0.032
Age (2020q1)	0.036***	0.036***	0.015	0.015
Age^2^ (2020q1)	-0.000***	-0.000***	0.000	0.000
Female	0.085***	0.085***	-0.092**	-0.092**
Number of prescribed medications in the previous quarter	0.019***	0.019***	-0.002**	-0.002***
Observations	69,193	69,193	23,263	23,263
BIC	219,381.6	219,365.5	50,500.1	50,508.8
Model (link function$g$)	Negative Binomial		Linear	

Significance levels: ***0.01; **0.05; *0.1; *BIC* Bayesian-Information-Criterion; coefficients of other control variables and the trend specification are not shown due to space considerations but can be found in Table S2

In Table 6, the estimated pandemic outbreak effects ($\delta$) are shown for different trend specification parameter $P$. For both outcomes, the pandemic effect is rather robust to the trend specification. However, the estimated decline in practice visits varies from 26.0% for the linear specification ($P=1$) to 11.5% for $P=7$. The pandemic effect for the HbA1c-value is insignificant for all trend specifications.

**Table 6:** Sensitivity analysis

Trend specification:$P$		1	2	3	4	5	6	7	8
Number of practice visits	$\delta$	-0.260***	-0.117***	-0.161***	-0.176***	-0.192***	-0.157***	-0.115***	-0.122***
	BIC	219,758.1	219,552.9	219,421.6	219,431.4	219,400.4	219,388.3	219,391.7	**219,381.6**
HbA1c-value	$\delta$	0.013	-0.002	**-0.028**	-0.04	-0.035	-0.036	0.018	0.012
	BIC	50,502.8	50,510.9	**50,500.1**	50,509.5	50,510.5	50,510.5	50,512.2	50,510.8

δ: effect of the pandemic outbreak; *BIC* Bayesian Information Criterion; significance levels: ***0.01; **0.05; *0.1

## Discussion

The described development of an infrastructure for the analysis of pseudonymized routine data extracted from the EHRs of private primary care practices has created an opportunity to evaluate routine data of ambulatory service providers in a timely manner. Based on positive ethical votes and contractual regulations, routine data are thus available for research for the first time in Germany without any time delay, offering the analysis of diagnoses, laboratory results and prescriptions and, if applicable, the free entries of the physicians.

The results of this analysis clearly showed the benefits of routine data. Without this option of evaluating the routine data, the overview of the multimorbidity at the level of the individual chronic disease would have been a separate, cost-intensive and time-consuming study [30]. The participation rates in the DMP program found in this study allow the start of a new approach to quality management, as there are bias-free data on this topic compared to a study based on primary data [31]. Research questions to identify reasons which patient is enrolled in a DMP or not could shed new light on this proactive form of care [32].

Additionally, the developed infrastructure offers the development and application of automated algorithms (e.g., based on artificial intelligence) that could be used for automated clinical decision support [33], since the results of the externally analyzed exported routine data from the EHR could be reimported into the EHR. For instance, the relationship between prescribed medication, laboratory results, body weight, recorded diagnoses and frequency of medical consultations could be externally evaluated and reintegrated in the EHR to identify high-risk patients. The EHR could show a special marker or pop-up screen to inform the treating physician of the potential high-risk constellation. The imported results could be used to automatically create a patient recall lists in the EHR for scheduling a consultation. Further, the CSV import file in the described infrastructure enables the import of further non-medical data sources into the EHR, as complex online questionnaires from an external tool. This would enable analyses based on a combination of primary and secondary data.

The effects of the pandemic on clinical parameters are inconsistent, e.g., because of poorly controlled blood pressure values. However, these changes are already comprehensible before the pandemic and should therefore be further investigated in follow-up studies. Next steps should include the complementary performance of qualitative studies to determine the more precise reasons how the quantitatively observed change occurred [34, 35].

No pandemic effect on HbA1c levels was found for patients with DM type 2 disease. A similar conclusion was reached by a routine data analysis of 32,399 patients with DM type 2 based on the "Disease Analyzer Database", which comprises a representative panel of medical practices in Germany providing anonymized patient data from routine care. The data show a 0.04% increase in HbA1c from 2019 to 2020 [36]. A meta-analysis with a total of 1823 DM type 2 patients from eight included studies concluded that the lockdown had led to a short-term worsening of glycemic parameters in patients with DM type 2 [37]. Similar findings were observed in another meta-analysis. Based on data of 16,895 patients the levels of glycated hemoglobin increased by 0.34 [38]. While others observed an increased in the BMI after the lockdown [37, 39], in our data the weight of the patients remain unchanged.

The reduced utilization behavior due to the Covid pandemic was expected to disrupt the continuous primary care of patients with DM type 2 disease. As a consequence, the monitoring of blood glucose metabolism to minimize diabetic sequelae could be limited leading to more hospital admissions. However, our and other studies’ data do not support this supposition. In this study, the referrals to hospital as well as metabolic derailments have not changed or have even decreased among patients that were enrolled in a DMP. Based on data extracted from the French National Hospital Discharge database, the number of hospitalizations for diabetic foot ulcers also decreased [40].

### Strengths and limitations

The study highlights the benefits and potential of analyzing routine data extracted from the EHRs of ambulatory care practices. Regarding the empirical application of evaluating the effect of the outbreak of SARS-CoV-2 on the utilization of primary care services for patients with type 2 diabetes mellitus.

The study has strengths as well as limitations. A strength of this study is that it relies on routine data collected for all patients of practices in a specific region of Germany. Therefore, there are no issues related to any selection or response bias, as might be the case when primary data are used [41]. Social desirability bias might play a particular role when surveying patients about their health care utilization. However, given the free provider choice in Germany, we cannot take into account, whether the identified patients have additionally utilized primary care services at other practices. This problem might lead to underestimated figures of the DMP enrolment rate, since we cannot control, whether a patient is enrolled in another practice. Future research is needed to provide a general definition of a primary care patient under free provider choice that can be used in similar routine data analyses.

The focus on specific regions of the federal state of Baden-Württemberg constrains the representativeness of the findings. However, in contrast to routine data extracted from statutory health funds (that are accessible for researchers) the data from this study are not restricted to statutorily insured patients that cover 84% of the population of Baden-Württemberg [42]. Further, the data include all information about the treated patients that are deposited in the EHR. Most studies based on routine data of ambulatory health care services cannot combine information about the diagnoses, medication, laboratory findings or anamnesis. This unique data set of this study therefore offers a wide range of research opportunities that cannot be addressed by other data sets in Germany.

## Conclusion

The study has shown that the analysis of routine data from outpatient care in Germany is possible in a timely manner. The infrastructure established offers the potential for further analyses as well as the application of automated algorithms in the future.

The multivariate analyses show that there was a significant negative effect of the SARS-CoV-2 outbreak on utilization of outpatient services of patients with DM type 2 disease. This decrease was less pronounced among DMP patients. Two years after the start of the Covid pandemic the decreased utilization has not significantly worsened or improved the course of illness of the considered chronically ill patients. However, it must be taken into account that the observation period for clinically relevant outcomes is still relatively short.

## Supplementary Information

**Additional file 1: Table S1.** Number of practice visits and HbA1c of patients with DM type 2 per quarter.** Table S2.** Full estimation results of the multivariate analysis of the Covid-Outbreak.

## Data Availability

The datasets generated and analyzed during the current study are not publicly available due to strict German data protection regulations, the legal provisions with the MEDI-Verbund Baden-Württemberg e.V. and the guidelines of the ethics committee of Luebeck University. However, data are available from the corresponding author on reasonable request with the permission from the MEDI-Verbund Baden-Württemberg e.V.
