# Supplementary material for: The use of routine data from primary care practices in Germany to analyze the impact of the outbreak of SARS-CoV-2 on the utilization of primary care services for patients with type 2 diabetes mellitus
Source: BMC Prim Care. 2022 Dec 16;23:327. doi: 10.1186/s12875-022-01945-y (PMC9754999; doi:10.1186/s12875-022-01945-y)
Supplement: Supplementary file 1 — Additional file 1: Table S1. Number of practice visits and HbA1c of patients with DM type 2 per quarter. Table S2. Full estimation results of the multivariate analysis of the Covid-Outbreak. [file 12875_2022_1945_MOESM1_ESM.docx]

**Appendix**

**Table S1: Number of practice visits and HbA1c of patients with DM type 2 per quarter**

|  |  | Number of practice visits | | HbA1c (%) | |
| --- | --- | --- | --- | --- | --- |
|  |  |  | DMP |  | DMP |
| year | quarter | /n* | /n* | /n* | /n* |
| 2016 | 1 | 1.24/4182 | 1.55/2467 | 7.04/733 | 7.07/637 |
| 2016 | 2 | 1.22/4181 | 1.54/2466 | 7.03/739 | 7.04/638 |
| 2016 | 3 | 1.17/4174 | 1.47/2462 | 6.86/715 | 6.89/630 |
| 2016 | 4 | 1.18/4163 | 1.50/2455 | 6.95/742 | 6.99/643 |
| 2017 | 1 | 1.18/4140 | 1.55/2442 | 7.02/610 | 7.05/543 |
| 2017 | 2 | 1.19/4137 | 1.54/2441 | 7.01/798 | 7.04/688 |
| 2017 | 3 | 1.19/4131 | 1.51/2440 | 6.90/795 | 6.94/695 |
| 2017 | 4 | 1.24/4120 | 1.59/2434 | 6.94/823 | 6.95/716 |
| 2018 | 1 | 1.49/4095 | 1.91/2418 | 6.99/865 | 7.03/749 |
| 2018 | 2 | 1.41/4091 | 1.80/2416 | 6.87/964 | 6.90/834 |
| 2018 | 3 | 1.44/4081 | 1.85/2408 | 6.84/1056 | 6.85/932 |
| 2018 | 4 | 1.47/4072 | 1.89/2403 | 6.88/1413 | 6.89/1246 |
| 2019 | 1 | 1.71/4043 | 2.14/2385 | 7.03/1418 | 7.06/1226 |
| 2019 | 2 | 1.66/4038 | 2.14/2383 | 7.00/1438 | 7.03/1247 |
| 2019 | 3 | 1.63/4028 | 2.07/2378 | 6.89/1390 | 6.92/1193 |
| 2019 | 4 | 1.54/4004 | 1.96/2359 | 6.93/1531 | 6.97/1334 |
| 2020 | 1 | 1.79/3958 | 2.17/2334 | 7.1/1414 | 7.16/1142 |
| 2020 | 2 | 1.37/3957 | 1.64/2333 | 7.11/1205 | 7.17/1006 |
| 2020 | 3 | 1.75/3947 | 2.15/2326 | 6.96/1603 | 6.99/1315 |
| 2020 | 4 | 1.38/3930 | 1.65/2317 | 6.99/1540 | 7.06/1237 |
| 2021 | 1 | 1.3/3849 | 1.52/2273 | 7.05/1597 | 7.15/1276 |
| 2021 | 2 | 1.23/3842 | 1.48/2269 | 7.00/1449 | 7.07/1160 |
| 2021 | 3 | 1.24/3823 | 1.50/2254 | 6.98/1368 | 7.03/1099 |
| 2021 | 4 | 1.24/3801 | 1.45/2243 | 7.01/1381 | 7.09/1117 |
| 2022 | 1 | 0.93/3731 | 1.15/2203 | 6.99/1279 | 7.08/1008 |

^a^Number of available observations in the respective quarter

**Table S2: Full estimation results of the multivariate analysis of the Covid-Outbreak**

| Variable | Utilization:  Number of practice visits | | Course of the disease:  HbA1c | |
| --- | --- | --- | --- | --- |
| *Trend specification* |  |  |  |  |
| $t$ | 0.627* | 0.613 | -0.039*** | -0.039*** |
| $t^{2}$ | -0.316** | -0.310** | 0.004*** | 0.004*** |
| $t^{3}$ | 0.073** | 0.072** | -0.000*** | -0.000*** |
| $t^{4}$ | -0.009** | -0.009** |  |  |
| $t^{5}$ | 0.001*** | 0.001*** |  |  |
| $t^{6}$ | -0.000*** | -0.000*** |  |  |
| $t^{7}$ | 0.000*** | 0.000*** |  |  |
| $t^{8}$ | -0.000*** | -0.000*** |  |  |
| *Quarter (Reference: Quarter 1)* |  |  |  |  |
| Quarter 2 | -0.023* | -0.023* | -0.060*** | -0.060*** |
| Quarter 3 | 0.029*** | 0.030*** | -0.134*** | -0.135*** |
| Quarter 4 | 0.003 | 0.003 | -0.097*** | -0.097*** |
| *Pandemic & DMP* |  |  |  |  |
| Covid-Outbreak ($\delta$) | -0.122*** | -0.189*** | -0.028 | -0.001 |
| DMP | 0.450*** | 0.407*** | 0.303*** | 0.318*** |
| Covid- Outbreak ($\delta$) X DMP |  | 0.092*** |  | -0.032 |
| *Socio-Demographics* |  |  |  |  |
| Age (2020q1) | 0.036*** | 0.036*** | 0.015 | 0.015 |
| Age² (2020q1) | -0.000*** | -0.000*** | 0.000 | 0.000 |
| Female | 0.085*** | 0.085*** | -0.092** | -0.092** |
| Number of prescribed medications in the previous quarter | 0.019*** | 0.019*** | -0.002** | -0.002*** |
| Practices-ID (Reference: ID 1) |  |  |  |  |
| 2 | -0.030 | -0.030 | -0.054 | -0.054 |
| 3 | -0.016 | -0.020 | -0.078 | -0.078 |
| 4 | -0.060* | -0.062** | -0.077 | -0.077 |
| 5 | -0.048 | -0.053 | -0.022 | -0.021 |
| 6 | -0.089** | -0.093** | 0.066 | 0.067 |
| 7 | -0.007 | -0.006 | 0.039 | 0.039 |
| 8 | -0.084** | -0.088** | 0.098 | 0.098 |
| *ICD-Chapters (Nr., ICD-10-Codes)* |  |  |  |  |
| I (A00-B99) | 0.025* | 0.025* | -0.027 | -0.027 |
| II (C00-D48) | 0.061*** | 0.060*** | -0.018 | -0.018 |
| III (D50-D90) | 0.074*** | 0.074*** | -0.049 | -0.049 |
| IV (E00-E90) | 0.062*** | 0.061*** | -0.030*** | -0.030*** |
| V (F00-F99) | 0.050*** | 0.050*** | -0.039* | -0.039* |
| VI (G00-G99) | 0.058*** | 0.057*** | -0.035** | -0.035** |
| VII (H00-H59) | 0.018 | 0.019 | 0.027 | 0.026 |
| VIII (H60-H95) | 0.088*** | 0.089*** | 0.004 | 0.004 |
| IX (I00-I99) | 0.052*** | 0.052*** | 0.014 | 0.014 |
| X (J00-J99) | 0.019 | 0.019 | 0.013 | 0.013 |
| XI (K00-K93) | 0.024* | 0.024* | 0.020 | 0.020 |
| XII (L00-L99) | 0.060*** | 0.061*** | -0.041* | -0.041* |
| XIII (M00-M99) | 0.048*** | 0.047*** | -0.003 | -0.003 |
| XIV (N00-N99) | 0.065*** | 0.065*** | -0.024 | -0.024 |
| XV (O00-O99) | 0.298 | 0.285 | -0.021 | -0.016 |
| XVI (P00-P96) | -0.107 | -0.102 | - | - |
| XVII (Q00-Q99) | 0.013 | 0.015 | -0.054 | -0.055 |
| XVIII (R00-R99) | 0.079*** | 0.079*** | -0.015 | -0.015 |
| XIX (S00-T98) | 0.089*** | 0.089*** | 0.006 | 0.006 |
| XX (V01-Y84) | 0.292** | 0.291** | -0.141 | -0.141 |
| XXI (Z00-Z99) | 0.079*** | 0.079*** | -0.012 | -0.012 |
| XXII (U00-U99) | 0.014 | 0.013 | -0.018 | -0.018 |
| Observations | 69193 | 69193 | 23263 | 23263 |
| BIC | 219381.6 | 219365.5 | 50500.1 | 50508.8 |
| Model (link function$g$) | Negative Binomial | | Linear | |

Significance levels: ***0.01; **0.05; *0.1; BIC: Bayesian-Information-Criterion.
